# Reflex Nervous Influence

**Published:** 1885-01

**Authors:** 


### REFLEX NERVOUS INFLUENCE.

It has oftentimes been cast up to physicians, by those who ought to know better, that the mysterious and ill-defined influence of "reflex action," is utilized as a shield to cover ignorance, and as a loophole to crawl through and escape when confronted with a morbid condition, the intimate nature and etiology of which they are unable to fathom.

That some men have availed themselves of this convenient and comprehensive term is undoubtedly true; but that such a thing as reflex action is a reality, and that it is a much more potent etiological factor of disease than is ordinarily believed, is also true.

By reflex action we mean that an impression made upon some nerve termination in one portion of the body is carried along this nerve to a center, and from there reflected, as it were, along some other nerve to a part of the body remote from the point of first impression, at which latter point its power to disorder healthy action is made manifest, while no morbid phenomena are observable at the point from which the irritation has really arisen.

This is a plain definition of "reflex action," devoid of all technical and superfluous words; and that diseased conditions frequently have such origin, no one of experience will deny.

But the general practitioner, we fear, does not take this factor sufficiently into consideration in the formation of his opinion of the cause of disease, and since, therefore, his remedies are directed rather to the effect than to the cause of the effect, he is met oftentimes with failure, when, did he but realize the actual influence of reflex action, and look to the proper point for his cause, and guide his therapeutics accordingly, he would have much better results.

